# Managing Emergency Situations in the Smart City: The Smart Signal

**DOI:** 10.3390/s150614370

**Published:** 2015-06-18

**Authors:** Ángel Asensio, Teresa Blanco, Rubén Blasco, Álvaro Marco, Roberto Casas

**Affiliations:** Aragón Institute of Engineering Research (I3A), Universidad Zaragoza, Edificio IDi, Mariano Esquillor s/n, Zaragoza 50018, Spain; E-Mails: ter@unizar.es (T.B.); rblasco@unizar.es (R.B.); amarco@unizar.es (Á.M.); rcasas@unizar.es (R.C.)

**Keywords:** *Smart Signal*, Smart City, wireless networks, tunnel

## Abstract

In a city there are numerous items, many of them unnoticed but essential; this is the case of the signals. Signals are considered objects with reduced technological interest, but in this paper we prove that making them smart and integrating in the IoT (Internet of Things) could be a relevant contribution to the Smart City. This paper presents the concept of Smart Signal, as a device conscious of its context, with communication skills, able to offer the best message to the user, and as a ubiquitous element that contributes with information to the city. We present the design considerations and a real implementation and validation of the system in one of the most challenging environments that may exist in a city: a tunnel. The main advantages of the *Smart Signal* are the improvement of the actual functionality of the signal providing new interaction capabilities with users and a new sensory mechanism of the Smart City.

## 1. Introduction

A signal is a visual representation that transmits particular information in a quick and intuitive way. Signals should be designed to rapidly capture the attention of people, being their iconography easily understandable and self-contained, in order to ensure that the message is immediately read without any misinterpretation. Usually signals transmit data related to the context in which they are located and offer information about how to interact with them. In some types of signaling, such as emergency signals, the transmitted information is critical, as it warns about potential risks or gives vital cautions about how to proceed to face or to avoid these risks. That is why signals are more common and necessary in complex or hostile environments, which should be approached with particular caution. Nonetheless, in these contexts, signaling systems are limited nowadays: most of them are constituted either by merely passive elements that keep their message always visible, or by some kind of lighting controlled by simple external events [1,2] (as the ambient light level, alarm systems, *etc*.).

Signaling systems are closely related to the cities, either to the infrastructure of the city itself, to the buildings and facilities that the city contains, or to the communication channels within the city. 

Together with the signaling systems, other typical systems on cities are those intended to light the streets or public spaces. Both share characteristics, because they are composed of a lot of devices scattered throughout the entire city, *a priori*, and are simple devices of which the citizens are not aware. Unlike lighting systems, which have seen increases in their performances and efficiency using new technologies [3,4], the same has not happened with signaling systems. So, one of the aims of this paper is to show the possibilities that arise when performing a similar process in the field of signaling.

Currently, new capabilities are being included to the basic model of signal. Thus, in the area of context awareness, innovation is mainly focused on traffic control systems [5,6] (even commercial solutions [7]) and context awareness signaling in hostile environments [8,9,10]. The user interaction is evolving through variable message panels, whose usefulness has been demonstrated, although some doubts about the induction of distractions on drivers have arisen [11].

From the perspective of ICT (Information and Communications Technologies), it is common to use the term Smart City [12] to refer to the “city of the future” [13]. The current development of smart cities is in the early stages [14,15]; most of the services currently developed, are based on monitoring or sensing variables to extract information, analyze and show it to the user in a more useful way. This applies to numerous scopes like traffic management, monitoring of pollution [16] and environmental variables, water supply analysis [17], public WiFi, citizen information services based on position, or systems for the preservation of historical heritage [18]. To a lesser extent, it is possible to find examples of control of some of the typical systems of a city such as street lighting systems [19], traffic management and even power management [20] (integrating the Smart Grid inside the Smart City). There are many lines of work around the concept of Smart City and it is expected to become a sizable market, with projections of nearly $40 billion spent on smart-cities technologies by 2016. 

The concept of Smart City has a strong technological component as it is built by interrelated environments in which different embedded elements are able to interact among them, and with users who inhabit them, in order to generate new services. The so-called Internet of Things (IoT) is shown as one of its basic components, since it proposes that everyday objects are globally accessible from the Internet and integrated into new services (Internet of Services (IoS)), having a remarkable impact in our society. Next decade will be dedicated to the Future Internet that, with its components IoS and [21] IoT, will promote the development of the concept of Smart City.

Since an intelligent signaling system must necessarily be integrated into its environment; in the context of Smart City, such systems should be able to interact with those related to city management. From a technical perspective, it is necessary that the new *Smart Signal* is one of the things that will be part of the IoT. As in many other cases, incorporating a system into the Smart City, or a device into the Internet of Things, nowadays, is done by adding some ICTs (mainly communications, sensors or processing) to the original element. In this way, a *Smart Signal* will most be a traditional “signal”, to which some “smart” capabilities will be added.

When designing a signal, it is necessary also to analyze graphics design, semiotics, behavioral psychology of different people interactions with the user, *etc*. Much of these aspects have been overlooked in this paper, because they are away from the topic, but have been taken into account (thanks to the collaboration with the signaling company Implaser) when designing a concrete application.

Unlike other examples of systems that are evolving to be “smart”, the same trend is not seen in signals. This is because in any signaling system, the number of items is high and they are required to hava a minimal cost. On the other hand, a signal is something that goes unnoticed due to its message often being very simple, but these messages are usually very important, and at some time critical. Thanks to technological advances (miniaturization, economic cost and energy consumption requirements), and the current trends on smart things, the Smart Signal will be completely viable. The transition from a signal to a Smart Signal evolves through several states in each of the following aspects:

- Message: From *static* (always visible the same message), to *active* (its visibility is controllable through blinking, fading, *etc*.), and finally *adaptive* (one message or another according to different circumstances).
- Control: From *uncontrollable*, to *controllable* (it is possible to define the behavior of the signal externally), and finally *autonomous* (the signal has the capability of control itself).
- Communications: From *isolated* (the signal has no communications capabilities), to *point to point* (the signal can communicate with a control point), and finally *mesh* (the signal can communicate with any of the elements on its surroundings).
- Context: From *isolate* (the signal has no knowledge about its environment), to *context awareness* (the signal has full knowledge about its environment, and can accommodate to it).
- Energetic dependence: From *external dependence* (the signal must constantly maintain power), to *autonomous* (for a while), and finally *self-sufficiency* (the signal does not need any external energy support).

A true Smart Signal must be aware of its context, able to analyze the situation and to inform optimally at all times. It must have communication skills that enable its integration into the IoT and share information and interact with other signals, thereby forming a network of intelligent signaling. The displayed message should be manageable both from a local intelligence (each signal decides its own message), from a network intelligence (depending on the state of the environment that spans the signaling system and on other messages from each signal) or even from external signaling network commands. The advantages of an intelligent signal against the current signs are:

- Improvement on the messages sent to users (who will have more and better capacity for effective decision-making).
- Messages are adapted to the circumstances of each moment.
- Interaction with new recipients of information (e.g., vehicles on a road), which will automate the sending of information, and facilitate certain actions (such as driving).
- Providing lots of geographically distributed information to other systems in real time, enhancing their capacity for analysis, forecasting of situations and incidents management.
- Increased possibilities for control signaling systems from both internal sensors and from external systems.

In this article, the concept of *Smart Signal* is presented and Section 2 deepens in the design of each of its components, the desirable characteristics and associated implications. *Smart Signal* concept is not addressed, as a single element, but as a multi-agent system, which forms intelligent signaling systems able to interoperate within larger entities, such as the Smart City. From this conceptualization, Section 3 presents the architecture and implementation of an intelligent system. The concept of *Smart Signal* is generic and can have different applications, as there exists different kinds of signals. This paper presents an example using Smart Signals within a smart emergency signaling system for tunnels and large infrastructures. Finally, in Section 4, we present the tests in laboratory and field and the results associated.

## 2. Smart Signal Design Consideration

**Figure 1:**
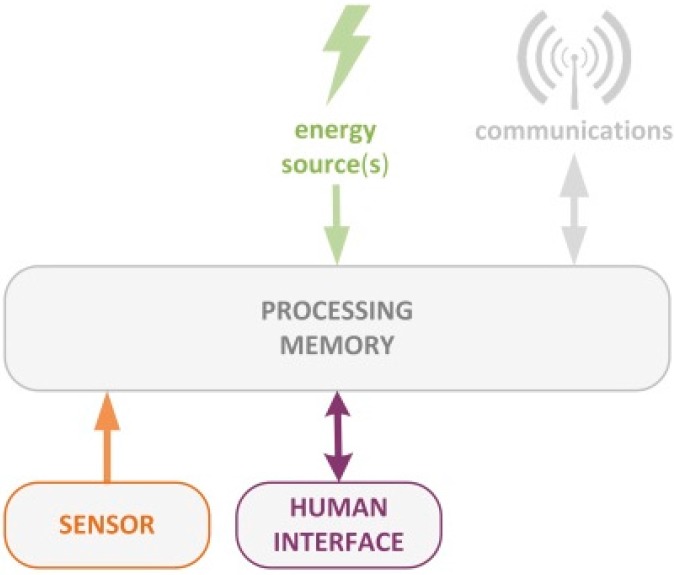
Smart Signal architecture.

According to state of the art, *Smart Things* are composed of different building blocks [22]. Figure 1 shows how this concept is particularized to the smart signal concept.

The processor is the core of the *Smart Things* devoted to making decisions, managing energy and communications, providing intelligence, providing context awareness, *etc*. The memory is a resource for the processor on which information is stored (settings, measured values, *etc*.). The following subsections detail each of the surrounding blocks that provide the Smart Signal’s functionality.

### 2.1. Human Interaction

Communication with users constitutes the main functionality of a signal. It must transmit to the people the message that the signal is intended for (for example, which direction to go in an emergency situation). Furthermore, smart signaling would aim to achieve a real human interaction, for example by getting user feedback (e.g., panic button in meeting point signal). 

Human interaction in smart signaling has to overcome a difficult environment that demands immediacy and effectiveness on a close to instantaneous basis. Simple and direct messages are mandatory, so that mainly elementary (but commonly used) output interfaces (lights, beeps, *etc*.) should be usually considered. Of course, the user must get the message meaning quickly and without any doubt, and appropriately decide the correct action, getting over a number of problematic situations in real environments, such as (i) a context with overwhelming information and stimuli; (ii) an unknown and stressful situation that sometimes even carries death risk; and (iii) an environment with communication blockings or interferences (for example, bad visibility of signals due to absence of electricity, hard to hear messages, difficulty to concentrate due to loud noise, *etc*.) 

A smart signal will be aware of its context, it will have external connectivity and intelligence to decide how to handle its user interfaces to get user attention and to transmit the appropriate message. The basic steps of human-signal interaction are: capture the attention, inspection, exchange of information, information processing, and decision-making. Considering them, along with the previous technical demands, here are the essential Smart Signal human–machine interface elements:

- Simple optical markers: Points or bright surfaces, both continuous and time-varying (blinking, flashing, strobe) with bounded chromatic variety (possibility of various colors, but without frequent changes).
- Simple panel displays with simple variables messages, and scarcely changing over time.
- Simple hearing devices able to synthetize voice or just sirens-like.

### 2.2. Context Awareness

A *Smart Thing* achieves context awareness through its sensors, which are intended to get information about the surrounding context (e.g., if close fire exists, if there are people around, *etc*.). This information can be used to rule the signal’s operation, to learn about the environment or just to report to a central management point. Sometimes, when we are interested in a magnitude inherent to the environment, a simple sensor can be used to measure it. In other cases, the *Smart Thing* must process the information of simple sensors (e.g., microphone) in real time in order to obtain higher level information (e.g., occupancy of a space through the analysis of the sound level monitored by a microphone). Therefore, it is relevant to identify what information from the context is required by a *Smart Thing*, which, obviously, will depend on its purpose. Focusing on the signals context, here we can see some of the variables that are susceptible to be measured, clustered by functionalities:

- Signals related with the ambient conditions. These kinds of signals measure one or more physical parameters of the environment and act according their value (e.g., a signal that monitors the temperature, “notifying about frost”, is enabled below $T_{Low}$ and disabled over $T_{High}$). These are the most usual parameters:
  ○ Temperature, humidity and pressure: They are used to notify about changes in the environmental conditions that may affect the road safety.
  ○ Wind: It can be used to notify the user about crosswind or other dangerous situations due to the wind.
  ○ Light/luminosity: A *Smart Signal* using this sensor can adjust its illumination level depending of the ambient light.
  ○ Volatile Organic Compound (VOC): It is used to determine the environmental pollution.
- Signals related with the detection of atypical environmental situations. These kinds of signals monitor atypical and dangerous situations (that should never happen in a normal situations), notifying to a central management point and/or acting, e.g., changing a traffic light, lowering a barrier, *etc*. Most usual parameters are water (to detect flooding), radiation, smoke or VOC (in order to notify that a security threshold has been overpassed), *etc*.
- User interaction signals. These kinds of signals notify users about their actions (e.g., speeding on a road, turning on the lights in a tunnel, *etc*.). Usually, the signal is inactive and, when a user action is detected, it starts showing an indication during a predefined time ($t_{Hold}$), after which, the signal returns to the inactive state. In some cases, the signal can be disabled for new detections for a time ($t_{Death}$) in order to avoid multiple activations). The most usual parameters for this kind of signal are speed, presence/movement, proximity, light/luminosity, zone trespassing (for example by crossing an IR barrier), *etc*.

All these parameters could be measured by simple sensors. Though, it is possible to find signals that need more advanced/abstract information, e.g., to detect a fire (by merging information from smoke, temperature and VOC sensors) or to identify a person on the road (by using cameras and artificial intelligence algorithms).

### 2.3. Communications

Today, the advances in wireless communication and batteries allows for distributing small devices with different degrees of intelligence in the context, so smart environments are used more and more [23], and it is possible to find applications in many different scenarios, such as the city [24], home [25] or country [26], and even, in signalization applications, such as the traffic management [27]. In all these fields, communications between the elements of the system are fundamental.

Communication implies not only the capacity to exchange data, but also implies understanding the information codified inside them. All the communication protocols ensure physical communication between things in the same network if sharing the same protocol; nevertheless, if the application layer is not defined, things will not be understood among them unless previously agreed between developers. So, once efficient connectivity between devices is granted, we can define standards (based on semantics and ontologies for complex information processing and support higher levels of abstraction) that provide interpretation to data exchanged between integrated sensors and actuators and the virtual world [28].

Each scenario has its own communication requirements (amount of exchange data, frequency of communication, existed or accessible infrastructure, number of devices, *etc*.) that determinate the selection of a specific protocol out of a set of protocols. We can find several wired (X10, CAM. Ethernet, Modbus, Profibus, *etc*.) and wireless [29] (Bluetooth, ZigBee, WiFi, GSM, *etc*.) standard protocols that facilitate interoperability between systems.

In the case of a *Smart Signal*, the typical application scenario could have an elevated number of elements (from a few tens to hundreds), separated (between tens to hundreds of meters) and heterogeneously distributed within a large scenario, such as a city. Although the loss of one of the elements of the system is not usually critical, it is unacceptable that this loss implies a segmentation of the communications of the system. Therefore, a mesh topology able to define alternative routes when an unforeseen event happens is an adequate solution. Regarding these scenario requirements, a WSAN (Wireless Sensor and Actuator Network) appears to be a suitable solution for the communication between the distributed elements. This situation produces coexistence between wirelesses and wired protocols because the WSAN usually ends with the interconnection to a fixed infrastructure (Internet) based on IP (Internet Protocol) [30]. Currently, technologies based on IEEE802.15.4, 6LoWPAN and IPv6 are combining to meet this demand [31,32].

### 2.4. Energy

All devices need energy to operate, and it can come from the mains, from batteries or harvested from the environment. Signals often have to operate in environments where it is not advisable to perform a wired installation, so energy efficiency becomes a key aspect in design. Therefore, when performing the electronic design of a *Smart Signal*, it will be critical to take into consideration different strategies of low power design [33,34,35,36,37].

Obtaining energy from the environment, or energy harvesting [38], is the process by which energy from a source environment is collected, processed and stored for future use by a standalone system [39]. Some new street lighting systems and beacon lighting systems, which are being developed for the Smart City, are beginning to incorporate these capabilities [40]. The most likely energy sources to be used in the field of signaling are light (either sunlight outdoors [41], or artificial light indoors [42,43]), heat, micro wind generators [44,45], movements generated by traffic [46] or systems that leverage weight [47,48] (surface pressure over devices on the floor) of vehicle pressure systems. Combining these sources of energy with secondary batteries is usually a suitable appropriate solution. 

In any case, power consumption of a *Smart Signal* will be a key factor to ensure its functionality; main sources of energy consumption are due to:

- Consumption associated with losses due to energy management (voltage conditioning, storage losses, *etc*.): $E_{Lost}$.
- Consumption associated with maintaining smart functionality (communication, sensing, data processing, *etc*.): $E_{SmartThing}$.
- Consumption associated with signaling: $E_{Signalling}$.
  (1) $$E_{Total}=E_{Lost}+E_{SmartThing}+E_{Signalling}$$

**Figure 2:**
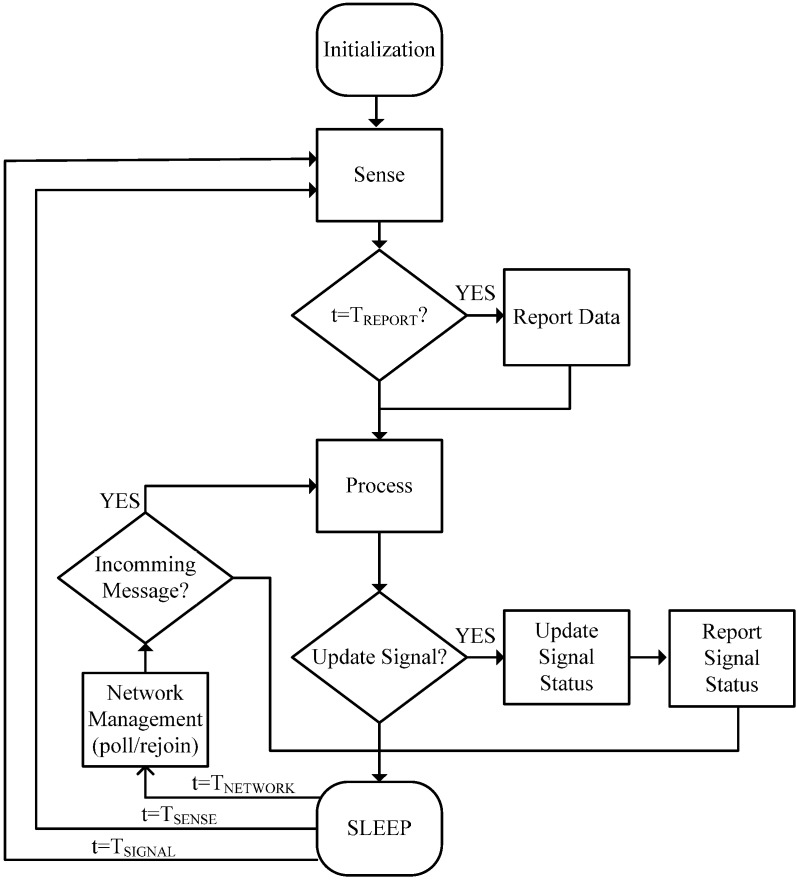
Operation cycle of Smart Signal.

To implement its functionality, after an initialization stage, the *Smart Signal* executes repetitively the same cycle, switching between active and sleep states (Figure 2). The standard cycle of a *Smart Thing* will be similar to a WSN node, and each of its states has an associated power consumption ($E_{state\_n}$):

- Sleep ($E_{SLEEP}$): This state is left, periodically or when an external event happens; the main causes for wake up are:
  ○ To stay (poll) or re-join the network, which runs every $T_{NETWORK}$.
  ○ To sense context, which runs every $T_{SENSE}$.
  ○ To refresh the status of the signal, which runs every $T_{SIGNAL}$.
  ○ Additionally, there may exist other causes, such as external events, that awake the system.
- Manage network status ($E_{NETWORK}$).
- Sense ($E_{SENSE}$): To get context awareness.
- Report Data ($E_{COMMDATA}$): To send collected data (each interval of time $T_{REPORT}$ that could be greater than $T_{SENSE}$), if applicable.
- Process ($E_{PROCESS}$): To analyze data, execute commands, set signal status, *etc*.
- Report Status ($E_{COMMSTATUS}$): To send, if applicable, new status of the signal.

The succession of these states, of predetermined time duration, is cyclically repeated, and defines the total $E_{SmartThing}$. This analysis is widely detailed in numerous papers, from which they can abstract the following considerations applied to *Smart Signal*:

- Consumption is periodic, with low average power consumption (in the order of the µW) that will mainly depend on the rate of time asleep/active.
- The higher peaks of power consumption (0.3 W–1 W) coincide with moments of communication.
- The need for keeping contact with the signal (to remain in the network) defines the $T_{NETWORK}$ time; periodically, the node will poll its parent asking for messages hold, and eventually will rejoin the network if the connection has been lost.

In short, the key to achieving low power consumption is to remain in a low power state most of the time. 

Moreover, considering the activation time of the signal, the following classification of its consumption, $E_{Signalling}$, is possible:

- Constant: The signaling is active all the time but the message can change according to circumstances and/or external references (as for signals that show environment conditions).
- Cyclic: Periods of activity and inactivity are alternated (as for beacon signals that operate only at night).
- Punctual: The signaling is activated only under special circumstances (as for a fire emergency signaling in a building).

Overall, while in the activation period, the consumption will be continuous with a higher mean average power (order of tens of W) than in the previous case. It is possible to use advanced strategies to modulate the power consumption while the activation period (an example is exposed later in this paper), in order to improve energy efficiency.

So:

(2) $$E_{SmartSignal}=E_{Initialization\_SmartThing}+N_{cycles}\times E_{Cycle\_SmartThing}+E_{Signalling}≃t_{total}\left(\left(1-D_{ST}\right)\times E_{SLEEP}+D_{ST}\times E_{ACTIVE}+D_{S}\times E_{Signalling}\right)$$

With:

(3) $$D_{ST}=\frac{t_{cycle\_SmartThing}-t_{sleep\_SmartThing}}{t_{cycle\_SmartThing}}$$

(4) $$D_{S}=\frac{t_{on\_Signal}}{t_{total}}$$

The issue of energy efficiency in a *Smart Signal* should be analyzed for each type of signal specifically, taking into account the environment in which it will be and the expected use. Given the dual nature of power consumption in a *Smart Signal*, it may be advisable to use two different energy sources for each of them, mainly to ease greater efficiency, but also for other reasons such as simplicity of the system, and cost. So, for example:

- For signals with few activations ($D_{s}↓↓$), it may use a separate primary battery to operate the “*signal*”, and use another strategy (the same or another battery, energy harvesting, *etc*.) to power the “*Smart Thing*”.
- If the activation signal is continuous ($D_{s}↑↑$), it is not feasible to use batteries primarily, but rather it is necessary to use a fixed power source ($E_{Signalling}\gg E_{SmartThing}$), whereas if the consumption related to the signal only is not excessive ($E_{Signalling}\times D_{s}↓$), using energy harvesting strategies for the entire system can be considered.

## 3. Smart Signal System Implementation

**Figure 3:**
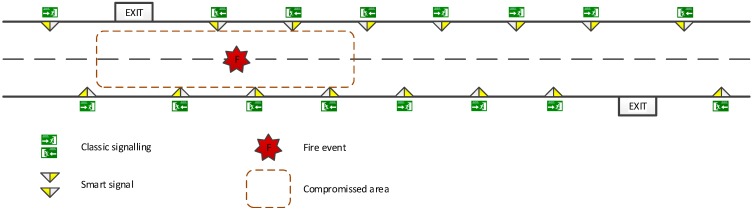
*Smart Signal* concept applied to emergency signaling in tunnels. The Smart Signals provide indications adapted to the emergency characteristics.

In order to demonstrate the capabilities of the proposed *Smart Signal*, this concept has been applied to emergency signaling, considering its use in a particularly complex scenario, such as tunnels or corridors inside buildings. Current systems have important limitations: they are closed and static, and they just passively inform the user about the nearest safety point. Therefore, we have designed a smart signaling system, in which the signals have mechanisms to transmit the message clearly and efficiently to users, adapted to both the circumstances of the signal environment and the reminder signals’ visualizations. The goal is to guide people through the most effective route and avoid particularly dangerous situation unlike traditional signaling: leading to exits that are compromised and dangerous. In the case depicted in Figure 3, the emergency (a fire and smoke) has compromised one of the emergency exits. Therefore, indications provided by some of the classic signals are unsafe, whereas the smart signals take this into account, and provide a safer indication.

The Smart Signals will be able to gather information from their nearby environment and to report it regularly, and it is also required that they integrate with the in-place management and control systems that monitor, control and manage the equipment already installed in the tunnel. The latter is achieved by means of an OSGi (Open Services Gateway Initiative)-based [49] gateway, which acts as entry point for the *Smart Signals* network, and also allows providing specific services, such as system management or information processing.

### 3.1. Smart Signal

The development of a Smart Signal can be addressed as if it were a node of a WSAN; this approach could simplify the process due to the know-how that already exists about them. Next, it will be exposed to the design of a *Smart Signal*, emphasizing the electronic design and user interaction.

#### 3.1.1. User Interaction

When considering the design of the proposed signal, the main specification was clear: to guide the people inside the tunnel to the closest and safest exit, the fastest way as possible. When an emergency situation occurs, different phases need to take place to solve it [50]: (i) detection, characterized by the time elapsing since the emergency happens to the moment the alarm is triggered; (ii) alarm, characterized by the time that takes all the warnings to be active; (iii) response, characterized by the time taken by the people to acknowledge the alarm and reaction to it; and (iv) movement, as the time needed to make it to safety. Signals play a key role in the third and fourth stages, as users should use them to orientate and evacuate themselves. A person needs to create a mental egocentric sequential map to follow; in this regard, signals can be essential way-finding beacons that must quickly help him/her to decide which direction to take, and then, as he/she advances, confirm whether or not his/her decision is correct [51].

In the specific case of a fire in a tunnel, the scenario becomes extreme; for this reason, emergency signaling has the function of directing pedestrians quickly to the nearest emergency exit. There is evidence (from previous accidents and simulations) that in emergency situations there is a percentage of people with a high reluctance to abandon their vehicles, which results in fatal outcomes for them, so they should be encouraged to leave the car for escape. The enormous burden of stress and bewilderment the user experiences in an event like this, which dramatically affects the ability to make clear decisions, is worsened by the existence of smoke. Apart from being an agent of intoxication, it makes the user become blind, thus, it is an important cause of confusion and disorientation. The evacuation phases (response and movement) are absolutely critical: signaling has to attract the user to the wall in order to offer him/her a benchmark; then, it has to transmit the information in a simple, direct, and effective way that is any free of dual meaning; and finally, it has to confidently make the user follow the safest way without delay or doubts. In summary, the aim of the signal is attracting the attention of the person, and convincing to him to leave his car and follow the escape indications without delay or doubt and as quickly as possible.

**Figure 4:**
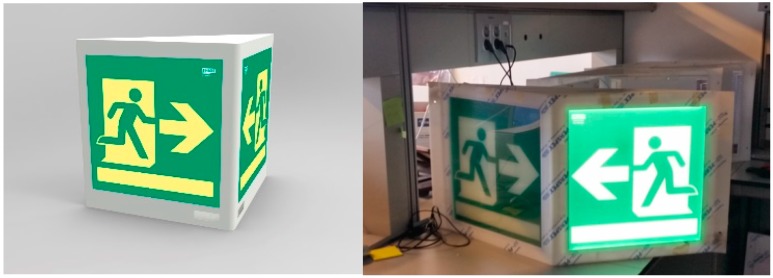
Render and signal prototype.

In our design for emergencies, signals are located in the walls of the tunnel and have a triangular prism shape. As in Figure 4, each of the faces of the prism will indicate opposite directions, but only one of them will illuminate in the event of alarm (central control will decide this). When a row of signals are installed in a tunnel separated by tens of meters, a user will see a flashing path of attracting green signals on one side and no lights at the other side. This effect is accentuated by the prismatic shape that conceals the unlit side; this constructive volume eases the lateral visibility (more common in a tunnel) and accompanies the correct orientation. This way, the signal’s design fulfills two main required functions: To attract attention indicating the right direction and reducing the response time and to confirm right direction as a person moves, clearing any doubts he/she should have and thus reducing movement time.

Our proposed implementation also considers the use of phosphorescent materials, due its obligatory use in different safety regulations [52]. Such materials allow capturing ambient light to ensure a minimum visibility in darkness, which makes the signal highly reliable, as it is a basic physical phenomenon. It should be noted that these materials present limited use when there is not enough ambient light to charge them, as in a tunnel environment. Accordingly, we propose using LED-based retro-illumination to periodically excite them, in order to keep minimum luminescence—in case of failure of the internal lighting—and to provide full intensity and maximum contrast in emergency situations [53].

**Figure 5:**
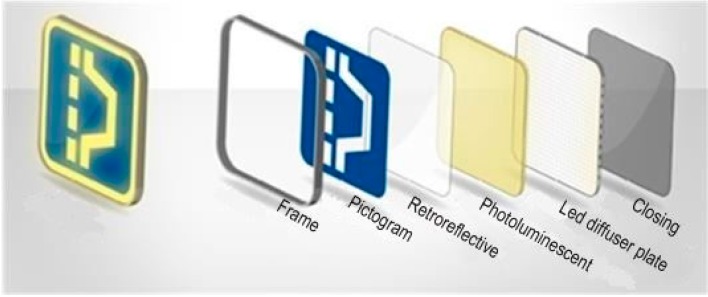
Signal layers scheme: pictogram, retro-reflective, photo-luminescent, and LED layers.

As in Figure 5, internally the signal has three layers: first there is a plate with the opaque mask with the pictogram’s negative; behind there is a sheet of phosphorescent translucent methacrylate; and then an LED panel backlight.

With this design, we control the energy transferred to the phosphorescent material, which will then be released in an uncontrolled but predictable manner. Thus, we go beyond regulatory guidelines, providing additional features such as remotely controllable dynamic messages, use in low lightning scenarios, energy efficiency that allow battery operation simplifying infrastructure, installation, and ensuring operation in emergency situations with no-electricity situations.

#### 3.1.2. Electronics

The Smart Signal itself is composed of two elements: a “traditional” signal (Figure 5) and an electronic device, which manages the signal. This electronic device has sensory, load control (used to handle the retro-illumination panels), communication and intelligent capabilities. This way, electronic and “traditional” signal become complementary, being possible to add Smart Thing capabilities to a signal previously installed or, even, to market them separately.

**Figure 6:**
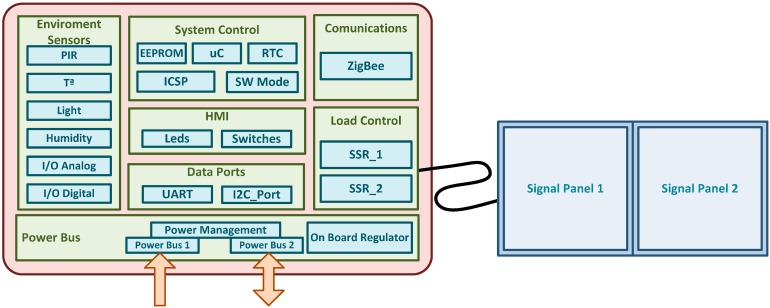
Block diagram of the electronic device.

In order to explain the electronic device design, it has been divided into several functional blocks (Figure 6). The next sections explain these blocks in more detail.

##### Power Management

This device has two power inputs, which enables to implementing different configurations.

1. Single power supply without backup battery: Power supply is provided through power bus 1 and power bus 2 is unplugged.
2. Single power supply with backup battery: Power supply is provided through power bus 1 and power bus 2 is connected to a backup battery, which is continuously charging. If the power supply is unplugged, the device is powered through the backup battery.
3. Double power supply: Power supply provided through bus 1 is used to power the electronic device and bus 2 is used to power the signal display.

Currently, the Smart Signal uses the last configuration, with a Lithium-ion battery (4.2 V 4.2 Ah) connected to power bus 1 and a battery pack of 12 V 14 Ah Alkaline Batteries (with a very low self-discharge current) connected to power bus 2 (this source is used only when a warning is detected).

##### Load Control

Load control block is used to turn on/off the retro-illumination panel (load). These panels are based in 12 V LED technology, with a peak power consumption of 8 W by panel. In this case, a SSR (Solid State Relay) has been selected as control element, in order to provide isolation between control and load, and also because it can switch on/off quickly, supporting different working modes (short or long blink, continuous lighting, *etc*.).

##### Sensors

Three sensors have been included in the PCB (Printed Circuit Board) design, all of them digital, simplifying the hardware design (as filtering and conditioning blocks are not required).

- Presence sensor: The PANASONIC EW—EKMC1601111, a PIR (Passive InfraRed Sensor), has been selected as presence sensor. This sensor activates an interruption when it detects movement inside their control zone.
- Light sensor: The APDS-9301-020 from AVAGO has been selected as light sensor. This sensor is managed by an I2C interface and can measure visible and IR irradiance in two separate channels.
- Temperature and humidity sensor: The SHT21 from SENSIRION integrates a temperature and humidity sensor in one device with a good performance and low cost. It is also controlled by an I2C interface. In situations where only the temperature measurement is required, it can be replaced by the cheaper LM75.

##### Control

The control block is performed by a microcontroller, an EEPROM memory and a rotatory switch:

- A low cost, low power, 8 bits microcontroller, the PIC18F26J11-I/SO from MICROCHIP has been chosen as control. Also, an external 31.876 KHz Xtal has been included in order to use the microcontroller’s internal RTCC (Real Time Calendar/Clock).
- To save the configuration parameters and other relevant data, a non volatile EEPROM of 8 Kbits is connected with the microcontroller by I2C.
- The rotary switch enables selecting one of the 10 preconfigured working modes.

##### Mesh Communications

Zigbee Communications have been implemented using the transceiver ETRX3 from Telegesis. This smart device follows a dual hardware architecture, where a low power microcontroller runs the application and controls a network co-processor implementing ZigBee Pro stack. After deep analysis and experimentation, we found this architecture more efficient in terms of power consumption than using a system on chip solution (embedding a radio module plus a programmable microcontroller), as it allows splitting tasks between two specialized microcontrollers; one for sensing tasks and the other for communication.

**Figure 7:**
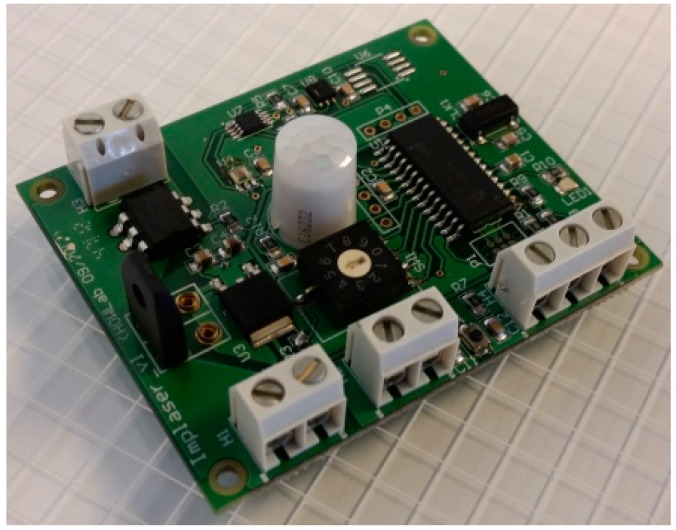
Electronic device.

To integrate all these capabilities, a specific hardware has been designed (Figure 7). The design of this device is aimed to allow for both its easy use in new *Smart Signals* and the adaptation of existing traditional signal. For this, several input/output (screw terminals for easy use), and items such as jumpers (for energy management settings) and switches (for the definition of operation modes) have been included. This electronic device has been successfully used to:

- Evaluate and show the different capabilities of a *Smart Signal* (as mentioned in Section 2.2. Context Awareness).
- Develop a solution to the problem of emergency management, as it will be seen later.

### 3.2. Gateway

**Figure 8:**
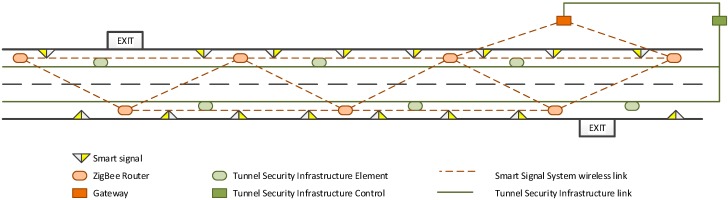
Smart Signal System infrastructure.

The *Smart Signals* communicate among them wirelessly through a mesh ZigBee network, backed by some dedicated, mains-powered devices, which route the messages exchanged (ZigBee Routers). Although in the proposed scenario, the detection of the emergency situations could be accomplished by other smart devices that would raise an alarm and would broadcast it directly through the ZigBee network, triggering of alarms has been delegated to the current tunnel security infrastructure (Figure 8).

When any of the Tunnel Security Infrastructure Elements (like smoke detectors, fire detectors, cameras, *etc.*) identifies a dangerous situation, it transmits that information to the Tunnel Security Infrastructure Control. There, the system itself or the operator in charge may decide to raise an alarm, which is delivered to the *Smart Signal* System through the Gateway (as well as to any other tunnel security infrastructure elements). The Gateway will broadcast the alarm message to the network, so the *Smart Signals* are notified about the emergency and react consequently. The Gateway is also in charge of typical system maintenance tasks, such as creating the ZigBee network, *Smart Signal* discovery and recruitment, general system health monitoring, *etc*.

**Figure 9:**
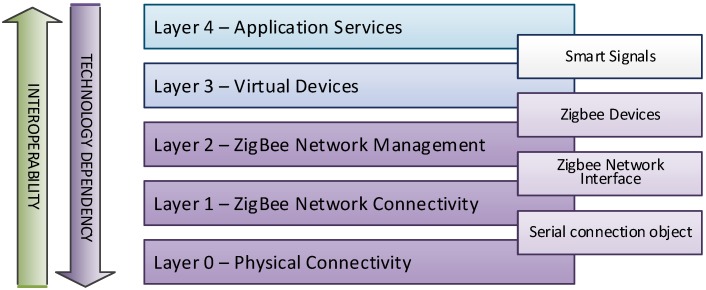
Gateway software architecture.

It is possible to find different proposals centered on the concept of Gateway [54]. In our case, we have used an embedded PC running Linux and a Java virtual machine where we have deployed an OSGi framework to implement the Gateway intelligence. Using OSGi as development framework eases development of the Gateway intelligence, as we can build modular software following the layered architecture depicted in Figure 9, and we may focus on one single layer when developing, while the whole application layers will be arranged at execution time. Each layer may comprise one or more bundles (isolated fragments of code that implement some behavior and are accessible to other bundles by publishing services in the OSGi framework).

The lower layer of the architecture is in charge of providing base connectivity with the ZigBee network through a USB dongle acting as network coordinator and network sink, which results in a sort of serial connection object allowing sending and receiving bytes as data streams. 

The second layer adds meaning to those bytes, implementing the specific communication protocol with the USB dongle and allowing effective communication with the ZigBee Network. This results in an object representing the ZigBee Network Interface (ZNI), which encapsulates the communication protocol with methods and fields.

The middle layer is in charge of managing the ZigBee network through the usage of the ZNI representation service, being able to discover ZigBee devices on the network, recruit them and handle network unavailability. At this layer, objects representing ZigBee devices are available, although they are already described in terms of the underlying technology.

In the next layer, objects related to ZigBee devices are described as Virtual Devices, regardless of their underlying technology, and whether they represent Smart Signals. 

The upper layer is devoted to Application Services, which use those Virtual Devices to provide the main Gateway intelligence. Specifically, there is a component (bundle) that offers a GUI allowing the operation of the Smart Signal System in a local fashion, and another component that creates a MODBUS slave and translates Smart Signals objects into a process image shared with the Tunnel Security Infrastructure Control, which behaves as the MODBUS master.

This architecture also allows easily adding other services, such as local processing of the information gathered by the Smart Signals (for example, with the temperature information provided by them, it would be possible to better characterizing the compromised area in fire-related alarms), or exposing that information not only through MODBUS, but other interfaces, without affecting existing functionalities.

## 4. Performance Evaluation

We performed evaluation of the system at various levels:

### 4.1. Lab Testing

**Figure 10:**
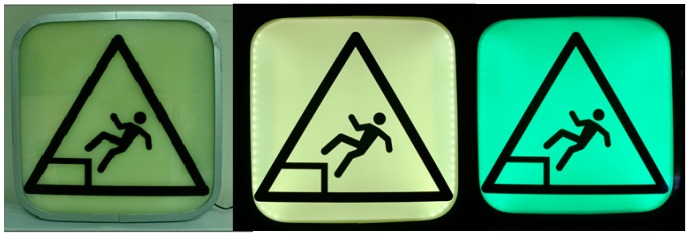
Uses of a *Smart Signal* with phosphorescent support.

Phosphorescent material can be considered a transitory repository of energy, similar to how batteries accumulate electrical energy. *Smart Signal* should wisely use its energy to provide required service with the minimum energy expenditure. Evidently, LED lighting will spend most of the energy, thus there is a number of important parameters to be decided in order to be the most efficient. We set up an experimentation bench to analyze signal performance measuring energy consumption and luminance variation in time. Figure 10 shows the panel used when it has no light source or luminescence, when illuminated using rear LEDs and when fully charged of phosphorescence.

Table 1 shows the parameters studied, the design of the experiment and the results of each experiment. 

**Table 1:** Parameters affecting the efficiency of Smart Signal with phosphorescent support.

Parameters	Experiment Design	Results
Wavelength and relaxation time	Excitation with white, blue and ultraviolet LEDs	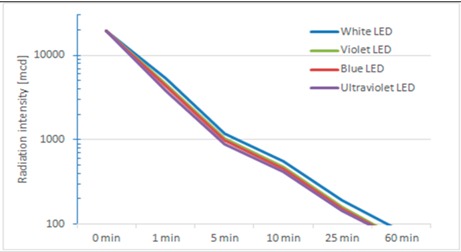 **Light source****Initial Radiation intensity (mcd)****Excitation time (s)****Electrical Energy Spent (joules)****Initial rad. Intensity performance (mcd/joule)****Radiated Intensity (cd·s)****Radiated Intensity performance (cd·s/joule)****White LED**19,70016020909.431276.360.61**Violet LED**19,700120842.423.331176.281.40**Blue LED**19,700120745.226.451150.191.54**UV LED**19,510180518.437.641093.542.11 We can observe that UV light requires the minimum energy to get same initial radiation intensity; around 25% of white and 70% of blue light. It can be also observed that although the initial radiation is roughly the same (19,700 mcd), the radiated energy provided during one hour is slightly larger for the white light This provokes that ratios of energetic performance of radiated energy in one hour to become better for white light, but still very far from UV.
Intensity and excitation time	Excitation of UV LED with different energy (variation of power and time) from 43 J to 518 J	**Voltage (V)****Excitation time (min)****Current (A)****Electrical Energy Spent (joules)****Initial Radiation intensity (mcd)****Initial rad. intensity performance (mcd/joule)****Radiated Intensity (cd·s)****Radiated Intensity performance (cd·s/joule)****12**10.24172.809720.056,25612.533.54**12**20.24345.6016,900.048.90973.042.82**12**30.24518.4019,510.037.641093.542.11**11**40.13343.2012,500.036.42852.682.48**10**40.08192.005182.026.99522.452.72**9**40.0243.20778.018.01100.582.33 It shows how lowering the radiated light power reduces the performance in terms of initial radiation. It happens similarly with radiated intensity but not so evident when saturating the luminescent material. Both evidences indicate that powering LEDs at nominal level is recommended and also exciting material little time.
Excitation frequency	Maintaining LED energy, modify excitation frequency from continuous to 100 Hz	**Frequency (Hz)****Excit. Time (min)****Electrical Energy Spent (joules)****Initial Radiation intensity (mcd)****Initial rad. intensity performance (mcd/joule)****Radiated Intensity (cd·s)****Radiated Intensity performance (cd·s/joule)**02345.6016,900.048.90973.042.8254345.609020.026.10669.721.94504345.606073.017.57549.831.591004345.605961.017.25550.971.59Spending the same energy but modulating it in different ways has a considerable impact in performance being continuous excitation the most efficient.

Considering that we have a primary battery reservoir of 12 V and 14 Ah and that we need 2 min of continuous excitation of UV LED consuming 345 joules (or white LED consuming 1567 joules) to get 10 min of acceptable luminescence, we can have 292 h of signaling (64 h with white LED). Thus, laboratory test confirmed the feasibility of a battery-operated signal that can guarantee a considerable level of visibility (much higher than traditional emergency signals) in an environment with little ambient light as a tunnel. 

### 4.2. Field Testing

For the evaluation of the system, several tests in the tunnel Monrepos I (roadway N330 of Autonomous Community of Aragon, Spain) have been conducted. The tunnel is a monotube tunnel with three traffic lanes, a length of 1449 m (a fith of Aragon by length), and built in the 1990s for improving access to the Aragonese Pyrenees. After being qualified as one of the least safe tunnels in Spain [55], it has undergone a security check and upgrade. In July 2014 (a few weeks before its reopening), a fully functional prototype of the proposed emergency signaling system was deployed. Previously, characterization tests for ZigBee wireless communications, which are not the subject of this article, has been done in the same tunnel.

**Figure 11:**
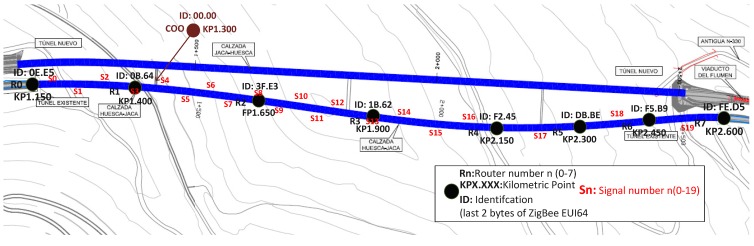
Distribution of routers and *Smart Signals* in Monrepos I tunnel.

The tunnel control center was emulated with a laptop controlling the rest of the system. This point was located in the first emergency exit tunnel (KP1.400); from there, the rest of the system was deployed (Figure 11) in an incremental way. This area was selected because it coincides with a curving stretch (one of the sections most complicated due to blocking the propagation of radio frequency). The rest of test setup consisted of:

- Eight routers: Separated approximately 250 m from each other, alternately placed in the wall on either side (preliminary tests showed that this was the best configuration).
- Twenty prototypes of *Smart Signal*: Separated approximately 50 m each other alternately placed in the wall on either side. So from 3 to 7 *Smart Signal* depended on each router*.*

**Figure 12:**
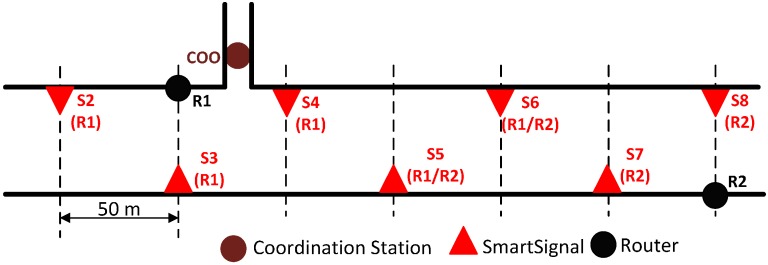
Details of setup around coordination station (scenario to test the perception).

Below (Figure 12) is a detail of the part of the deployment around coordinator station, corresponding with the section aimed at assessing the “signal element” and its interaction with the user:

When placing different elements of the system along the tunnel, the next distance must be considered:

- Distances between routers, $D_{Routers}$, will be such that for each deployment and RF settings (transmission power and antenna), in case a router falls, the infrastructure remains intact. This assumes that the minimum radius of coverage of each router is at least $2\times D_{Router}$.
- Distance between signals is defined by the applicable regulations. In the deployment shown, managers of the tunnel set the separation between signals at 50 m.
- Distance between signal and a router: The electronic design ensures that the minimum coverage of each signal is $D_{Routers}$, so in case a routers, the signal associated with it can immediately connect to the next.

For ensures correct distances, the RF characteristics of the devices are:

- Router: External ½ 50 Ω antenna (BKR2400 Embedded Antenna Design Ltd.), output power of 10 dBm and sensitivity of −106 dBm.
- Signal: On Board 50 Ω antenna (Ceramic Rufa Antenova), output power of 7 dBm and sensitivity of −100 dBm.

It should be noted that the maximum power of emission for European Compliance is 10 dBm (10 mW), and for FCC, IC Compliance is 20 dBm. The ZigBee transceiver used allows up to 22 dBm for routers and 8 dBm for signals.

The deployment process was carried out in the following steps:

1. Installation of coordinator station (emulator of the tunnel control center).
2. Installation of routers.
3. Establish communications infrastructure.
4. Placing prototype of *Smart Signal* near wall, alternately placed in the wall on either side.
5. Connecting and checking of signals connectivity. Fixing signals in wall.

**Figure 13:**
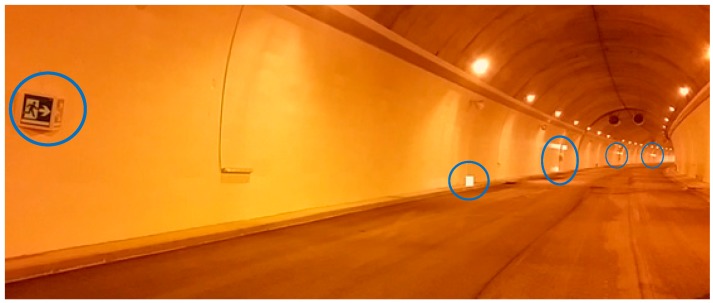
Snapshot during setup process. The closest signal (hanging on the wall) is turned off and the rest are turned on.

A similar process will be follow to deploy a definitive installation of the system. Figure 13 shows a picture of the real deployment of the system.

The proposed system is not aimed to detecting an emergency, but to improve the monitoring of the tunnel in a normal situation, and to provide additional information during an emergency situation.

- Temperature, humidity and light sensors report useful environmental information to the control staff of the tunnel in normal situations. In emergency situations, these parameters can provide additional information about the evolution of fire along the tunnel.
- Presence sensors allow for extracting information about the traffic along the tunnel in normal situations. Its main use is to detect activity in time bands outside the scheduled time of high traffic density. During an emergency situation, it is assumed that in certain environments this sensor will not be useful (as in fires, since sources of intense heat, or movement of thermal mass impede its operation), however it is very useful in proximity to emergency exits to estimate the level of influx of people.

In addition, the versatility of both the architecture and the *Smart Signal* allows for:

- On the one hand, to incorporate external sensors to the *Smart Signal* through the inputs provided for this purpose. During the deployment of the system, limit switch, proximity switch (for detecting emergency doors opened), and the external smoke sensors have been evaluated.
- Furthermore, to incorporate other devices into the system and take advantage of the communications infrastructure, we are currently working on the inclusion of a mobile sensor (and exploring its positioning capability).

Once the system was operating, various evaluation tests were conducted, focusing on the following aspects.

#### 4.2.1. User Perception Testing

The objective of these tests was to determine the validity of the final service offered by the system, abstracting from the technology. In a specific area of the tunnel, which we will call demonstration area, we carried out the following tests:

- Surveys to qualified staff (manager of the tunnel, maintenance operators, manager of a signalization company and expert in security from the Spanish Ministry) about their feelings of the system.
- Self-evaluation by the developers themselves.
- Simulation of user’s interaction with the signaling system. To this end, a group of users, underwent to the assessment of different predefined scenarios, in each of them:
  1. The evaluator placed one user at a point in the tunnel.
  2. The evaluator established secretly the location of a hypothetical emergency exit.
  3. The user is informed by the evaluator that there is an alleged fire and must find the emergency exit.
  4. The evaluator activates the signaling system and prompts the user to try to get to the hypothetical emergency door following the indications of emergency signaling system.
  5. During the progress of evaluation, the evaluator accompanied the user, logging their behavior and timing. At certain points in the tunnel (previously defined), the evaluator asks several questions (noninvasively with the test) about signal perception.

**Figure 14:**
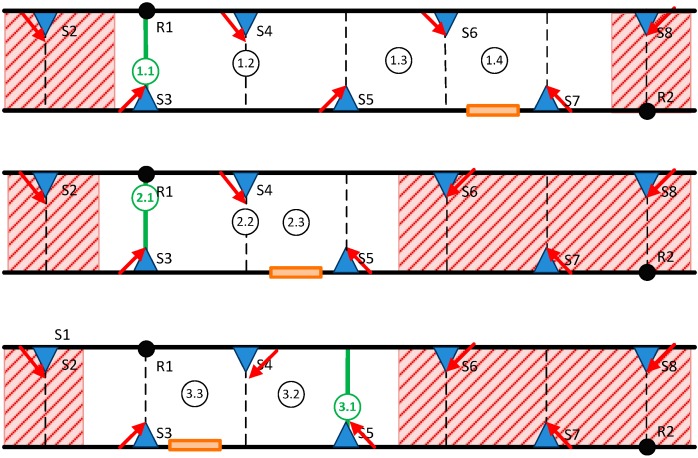
Examples of scenarios for simulating an emergency (in each of them, the emergency exit is a virtual point only known to the evaluator.

Thus, each of these trials consists of a path of the user accompanied by the evaluator, along the demonstration area. Seven situations were raised (with different parameters and circumstances). Figure 14 shows an example of three scenarios (1, 2, 3) evaluated. In each of them, there is the user’s start position (green circle X.1), and other positions where the user’s behavior must be analyzed and the evaluator registers the elapsed time. If the user enters the shaded area, a negative test result is considered.

In summary, we can say that all the tests about user perception resulted favorably, hence the system is considered a remarkable advance over existing signaling systems.

#### 4.2.2. Technical Testing

The objective of these tests is to characterize aspects of the technology used, which are impossible to evaluate in the laboratory (mainly regarding wireless communications in a tunnel). Various tests were conducted, and we have implemented a series of test benchmarks that run automatically in the coordinator station to analyze different aspect of the system. We discuss below the most important ones.

##### Routing and Behavior of the System When Communications Infrastructure Falls

**Figure 15:**
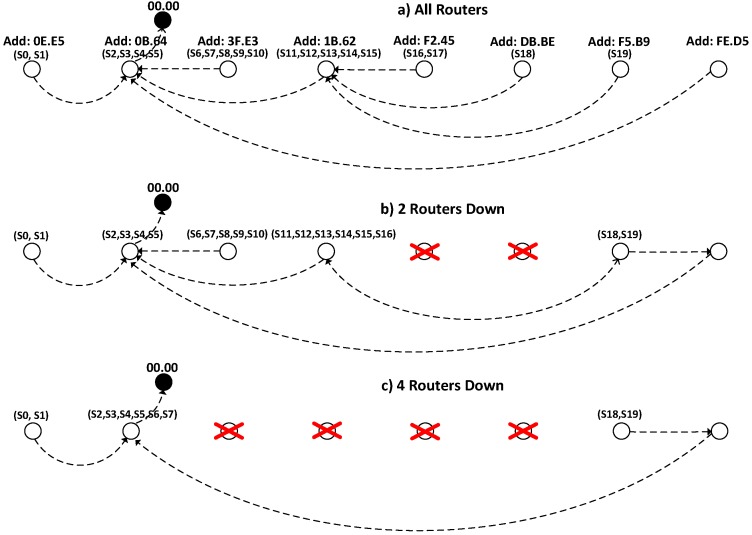
Routes and signals that depend on each router: (**a**) Initial snapshot; (**b**,**c**) simulating successive drop of nodes.

Routes are calculated automatically and dynamically, based on the best link power. A tunnel is a particularly complex environment, so previous studies were conducted to characterize the ZigBee communications. It is worth mentioning that the nature of this environment explains some unexpected behavior *a priori*, such as the link between nodes (FE.D5) and (0B.64) shown in Figure 15. The link is through a direct connection despite being the longest route, due to the topology of the tunnel (nearby routers to FE.D5 have the worst connectivity due to the curvature of the tunnel), and an area with electromagnetic noise in nearby routers was also detected. The road condition has been periodically monitored as a measure of network quality.

In addition, to evaluate the behavior in a failure scenario, the disconnection of several routers have been done simulating that they are destroyed. One advantage of the proposed system is its resilience, since in case of fire, although some of the routers fail (because they may be located in an area affected by the emergency), the system calculates the new routes, so unaffected areas remain operational. Additionally, as a measure of redundancy, the possibility of using several nodes prepared to adopt the role of coordinator could be contemplated, so that if the network is segmented, the system can still operate into independent networks.

##### Data Latency

**Figure 16:**
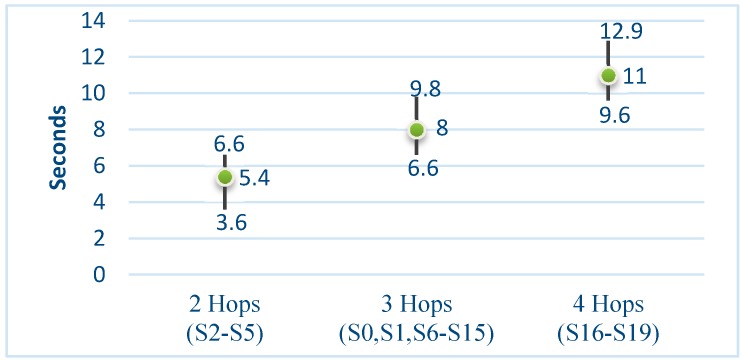
Latency intervals (within a 95% interval) of signals.

Message latency quantifies the availability of a *Smart Signal* in order to exchange information with it. We consider this availability from a total perspective (including all the layers), and define it, as how much time is required to force a *Smart Signal* to acknowledge a ping from the upmost layer of the *Smart Signal* Infrastructure. With this, latency depends on the following factors: processing time at the Gateway, number of hops between the gateway and the *Smart Signal*, time elapsed by the *Smart Signal* in Sleep mode, and the processing time in the Smart Thing. Both processing times are negligible compared with the other two parameters. Figure 16 shows the results, where we can see a direct relation between the latency and the number of hops.

##### Data efficiency 

It is common to consider the quality of a communication network as the relation between lost messages and total messages. For a global evaluation of the system, it is preferable to consider both ends of the system instead of point-to-point connections, thus we use the messages generated by the *Smart Signal* while sensing the environment *versus* the amount of data received in the gateway for a defined operation time. To calculate this value, we use only deterministic events (a scheduled predefined routine of monitoring in the Smart Signal) so the total data to be generated are well known *a priori*. In our case, the accumulated data efficiency is 95.3% (Table 2). It has been found that, in general, errors are due to unexpected problem with communications.

**Table 2:** Data efficiency.

**Number of Smart Signals**	20
**Smart Signal reporting rate**	1 data/min
**Operation time**	4 h
**Expected total messages**	4800
**Actual messages received**	4574 (95.29%)
**Messages lost**	227 (4.73%)

## 5. Conclusions

Signals are habitual elements present in cities. Therefore, it seems natural to consider them as important building blocks of the future Smart City. Moving from today’s signals (which are seen merely as passive and simple elements) to *Smart Signals* (with communication, sensing and improved user interaction capabilities), might turn the city’s signals into the main information channel of the Smart City.

However, to date, there is no proposal to use signals as main actors in the Smart City, nor to consider them as something with *more possibilities*. Thus, this paper presents the concept of the *Smart Signal* as the natural evolution of signaling in the context of the future Smart City, and as first class citizens on the Internet of Things ecosystem.

A conceptual model of *Smart Signal* inspired in the well-known model of *Smart Thing* has been proposed, highlighting the specific additional requirements related to signaling in terms of the interaction with the user. Based on this model, we have reviewed the technical features that a *Smart Signal* should have, mainly focusing on energy issues and wireless communications.

To validate the *Smart Signal* concept, an implementation in the context of a tunnel emergency signaling system has been presented, describing in detail the design and implementation of the *Smart Signal*, and its integration into a Smart Signaling System, as well as its deployment in a real setup, the Monrepos I tunnel (Aragón, Spain) in a fully operational way.

The *Smart Signal* and the Smart Signaling System have been evaluated exhaustively, both technically and functionally, evidencing its feasibility and its advantages over classical signaling. Actually, there is a perception that the system is very close to real use with a high chance that it will be turned into a commercial product.

The advantages of such *Smart Signal* concept are multiple. First, the actual functionality of the signal is significantly improved, and moreover new capabilities for interaction with users of the signals will be offered. Furthermore, there are some critical situations where there is no optimal solution currently, and in which a *Smart Signal* as the proposed could be an efficient and optimal solution. Second, *Smart Signals* could turn into the natural sensory mechanism of the Smart City, as communication and energy issues are the main constraints of Smart City objects, and aggregating sensing and signaling on the same component may significantly decrease the costs of Smart City infrastructure. Third, incorporation of *Smart Signals* to the Smart City may open the door to new services that will increase quality of life and safety of smart citizens.
